# The Impact of SARS-CoV-2 Infection on Sleep, Daytime Sleepiness, and Depression—Longitudinal Cohort Study

**DOI:** 10.3390/medicina60081352

**Published:** 2024-08-20

**Authors:** Klāvs Putenis, Sintija Strautmane, Madara Mičule, Evelīna Kočāne, Guntis Karelis

**Affiliations:** 1 Faculty of Medicine, Riga Stradiņš University, LV-1007 Riga, Latvia; klavsputenis@gmail.com (K.P.);; 2 Faculty of Residency, Riga Stradiņš University, LV-1007 Riga, Latvia; 3 Department of Neurology, Pauls Stradiņš Clinical University Hospital, LV-1002 Riga, Latvia; 4 Department of Neurology and Neurosurgery, Riga Stradiņš University, LV-1007 Riga, Latvia; m.lazdane@gmail.com; 5 Department of Neurology and Neurosurgery, Riga East University Hospital Clinic “Gaiļezers”, LV-1038 Riga, Latvia; 6 Department of Infectology, Riga Stradiņš University, LV-1007 Riga, Latvia

**Keywords:** SARS-CoV-2, COVID-19, sleep, sleep quality, daytime sleepiness, depressive disorder, fatigue, sleep wake disorders, quality of life

## Abstract

*Background and Objectives*: COVID-19 disease, caused by the SARS-CoV-2 virus, has presented significant challenges to global health, with acute and chronic implications for various aspects of well-being, including sleep and quality of life. This study aimed to investigate the impact of SARS-CoV-2 infection on sleep quality, daytime sleepiness, and quality of life in hospitalised and home-treated patients after three and six months. *Materials and Methods*: A longitudinal cohort study was conducted, enrolling hospitalised patients from a single clinical university hospital and home-treated participants through a survey spread through social networks. Individuals who had tested positive for the SARS-CoV-2 virus in the past three months and had a symptomatic course of the disease were included in the study. Participants with previously diagnosed sleep disorders were excluded from the study. Participants were evaluated using internationally validated self-evaluation scales, including the Epworth Sleepiness Scale (ESS), Pittsburgh Sleep Quality Index (PSQI), Patient Health Questionnaire-9 (PHQ-9) and Fatigue Severity Scale (FSS). Data were collected three and six months after laboratory-confirmed SARS-CoV-2 infection, with informed consent obtained from all participants. Statistical analysis was performed using the Wilcoxon signed rank test, Fisher–Freeman–Halton exact, Pearson Chi tests and Spearman correlation. Results were considered statistically significant with *p* value < 0.05. *Results*: In total, 66 participants with a mean age of 44.05 ± 21.61 years were enrolled in the study. Most patients (*n* = 36) were treated at home and 30 at hospital. Six months after SARS-CoV-2 infection, home-treated patients reported a higher prevalence of poor sleep quality (52.8%, *n* = 19, *p* = 0.015, PSQI) and hospitalised patients showed a lower prevalence of depressive symptoms (*p* < 0.001, PHQ-9) as 90% (*n* = 27) had minimal or no symptoms compared to 30.6% (*n* = 11) in a home-treated group. *Conclusions:* These findings mark the importance of the COVID-19 patients’ management settings as people treated at home had worse sleep quality and more depressive symptoms six months after infection indicating worse life quality.

## 1. Introduction

COVID-19 is an infectious disease caused by the SARS-CoV-2 virus. In 2019, it emerged as a global pandemic with significant health consequences [1,2,3].

Beyond the acute phase, SARS-CoV-2 virus infection caused massive global stress and many physiological effects consisting of a wide variety of long-term symptoms, called the post-COVID-19 condition (PCC) [3]. According to data available in the literature, psychiatric conditions, such as anxiety and depression, and cognitive dysfunction, are among the most observed physiological effects in PCC patients, including poor sleep quality [4,5].

Sleep is essential for preserving health, an important biological mechanism for maintaining homeostasis [6,7]. Sleep also significantly affects daily physical, mental, and social functioning and overall quality of life [6,7]. Sleep deprivation and reduced sleep quality have been associated with decreased daily functioning, cognitive decline, poor quality of life, and compromised immunity [6,7,8].

In the last three years, numerous studies have been conducted to analyse the effects of COVID-19 on sleep quality. Some publications focused on the effects of the pandemic and isolation on sleep quality, both in healthy subjects and among COVID-19 patients [9,10]. Other authors studied sleep disturbances in patients with COVID-19 [11,12].

Interestingly, Semyachkina-Glushkovskaya et al., 2021 published an article on brain mechanisms of COVID-19 sleep disorders reviewing findings and trends in sleep research from 2020 to 2021 [13]. They suggested that sleep disorders can induce neuroinflammation, affecting the blood–brain barrier with the subsequent entry of different antigens and inflammatory agents into the brain [13]. The phenomenon of ‘coronasomnia’, a sleep disorder linked to COVID-19 stress, is believed to be caused by anxiety and stress, depression, changing working hours, and prolonged use of social networks [14,15,16,17]. The phenomenon of ‘coronasomnia’ includes insufficient sleep, poor sleep quality, insomnia, sleep apnoea, disturbances in sleep–wake schedules, etc. However, blood–brain barrier leakage through neuroinflammation may also contribute to developing the phenomenon of ‘coronasomnia’ [13]. Semyachkina-Glushkovskaya et al., 2021 suggested that sleep hygiene and quality should be incorporated into the rehabilitation of COVID-19 patients [13].

While existing research has established a link between COVID-19 and sleep problems, there is a need for further investigation into the longitudinal impact of the disease on sleep quality, daytime sleepiness, and quality of life, particularly among individuals treated in different settings. This study aimed to address this gap by examining these outcomes in hospitalised and home-treated patients three and six months after SARS-CoV-2 infection. By comparing the experiences of individuals managed in different care settings, this study provides unique insights into the potential long-term effects of COVID-19 on sleep and quality of life.

Understanding these potential long-term effects on sleep can be invaluable for various specialists, informing future efforts to educate patients and healthcare professionals about the importance of sleep hygiene in managing post-COVID-19 complications and potentially, similar respiratory illnesses. The findings of this study contribute to the growing body of evidence on the impact of COVID-19 on sleep and highlight the need for ongoing research in this area.

## 2. Materials and Methods

### 2.1. Patient Selection

This prospective cohort study aimed to explore the longitudinal impact of SARS-CoV-2 infection on sleep, daytime sleepiness, and quality of life among individuals who had tested positive for the SARS-CoV-2 virus and had a symptomatic course of the disease. Participants were stratified into two groups based on the severity of their symptoms and subsequent treatment. The mild-symptom group consisted of individuals treated on an outpatient basis, while the moderate (there were clinical symptoms, which affected daily life activities, but were no radiological changes in the lungs, treated at high dependent unit), moderately severe (had lower airways inflammation with SpO_2_ > 94%, treated at high dependent unit) and severe (SpO_2_ was <94% or critical condition, treated at intensive care unit) symptom groups included patients treated at the hospital.

Inclusion criteria included patients above 18 years of age, laboratory-confirmed SARS-CoV-2 infection, comprehension, and ability to complete all the patient questionnaires. Exclusion criteria included previously diagnosed sleep disorders. All patients in this study signed a written informed consent form before participation, ensuring compliance with ethical standards and patient anonymity.

### 2.2. Data Collection

Recruitment of the participants started in April 2022 and continued until the summer of the same year, when the number of people infected by the SARS-CoV-2 virus rapidly decreased. The recruitment process for both groups started simultaneously. Participation in the study was completely voluntary, and no one received any compensation for participating.

#### 2.2.1. Hospital-Treated Participants

Upon discharge from the hospital, participants were informed about the opportunity to participate in this study. If they agreed, informed consent was obtained at that time. At the same time of recruitment, the respondents were asked to provide information on sociodemographic parameters, physical characteristics, clinical manifestations of acute infection, and vaccination status. Additionally, patients were requested to provide their telephone numbers for further communication. Other information about treatment, previous illnesses, and comorbidities was obtained from patient hospital documentation. Patients who met the inclusion criteria were interviewed by telephone at three and six months after they had tested positive for the SARS-SoV-2 virus. The same trained interviewer in Latvian conducted all interviews. The interviewer used validated self-evaluation scales in Latvian, as follows: the Epworth Sleepiness Scale (ESS) [18], Pittsburgh Sleep Quality Index (PSQI) [19], Patient Health Questionnaire-9 (PHQ-9) [20] and Fatigue Severity Scale [21] (FSS).

#### 2.2.2. Home-Treated Participants

Individuals treated at home were recruited through various social media platforms using an 18-question survey to collect demographic and clinical information analogous to that obtained from hospital-treated participants. All the information obtained was self-reported by the participants. Upon opening the electronic survey, respondents had the opportunity to familiarise themselves with the study design and give their consent to participate. Participants who met the inclusion criteria received an email with the second survey three and six months after being tested positive for the SARS-CoV-2 virus. The second survey had 58 questions in the Latvian language based on the same validated self-evaluation scales used for hospital-treated participants. The layout of the study and the questions stayed the same between the three- and six-month periods.

### 2.3. Study Variables

The primary interest included the results of ESS, PSQI, PHQ-9, and FSS. These instruments were administered to assess daytime sleepiness, sleep quality, depressive symptoms, and fatigue severity, respectively. Additional variables of interest included the severity of clinical manifestations, vaccination status, comorbidities, and duration of infection (for hospitalised patients refers to the time spent in a hospital while there is an active infection and for participants treated at home this refers to time since getting laboratory-confirmed positive SARS-CoV-2 test and when there are no symptoms of disease), and smoking status.

### 2.4. Data Analysis

Statistical analysis was performed using IBM SPSS Statistics Version 29.0.0.0. The descriptive statistics summarise the study population’s demographic characteristics and baseline scores. Wilcoxon signed-rank tests were used to compare changes in ESS, PSQI, PHQ-9, and FSS scores within each study group over time. Fisher–Freeman–Halton exact and Pearson Chi-square tests were used to find a significant association between the hospital and home groups with ESS, PSQI, PHQ-9, and FSS scores. Spearman correlation was used to extract the correlation between these health assessment tools.

## 3. Results

### 3.1. Patient Demographics and Characteristics

In total, 134 home-treated and 33 hospital-treated participants were selected for the study. After six months, 66 participants completed all necessary surveys and were enrolled in the study, and 54.5% (*n* = 36) were treated at home.

The study population was predominantly female, 74.2% (*n* = 49). The mean age was 44.05 ± 21.61 years, the mean body mass index was 25.79 ± 5.84 kg/m^2^ (Table 1). For 16.67% (*n* = 11) of the respondents, this was the second time they were infected with the SARS-CoV-2 virus. Relevant comorbidities included arterial hypertension (24.2%, *n* = 16), chronic heart failure (16.7%, *n* = 11), diabetes mellitus (13.6%, *n* = 9), and coronary heart disease (10.6%, *n* = 7).

Hospital-treated patients were significantly older and had considerably more comorbidities compared to the home-treated patient group (Table 1), *p* < 0.001. Among hospital-treated participants, 46.7% (*n* = 14) were not vaccinated; they had a more severe clinical manifestation; therefore, serious therapy was needed compared to 2.8% (*n* = 1) of unvaccinated individuals in the home-treated patient group where oxygen and drug therapy were not required (Table 2).

The duration of hospital treatment varied depending on the severity of the COVID-19 manifestation. Patients with moderate symptoms had an average hospitalisation of 8.29 days. Those with moderately severe manifestations required longer stays, averaging 18.33 days. Interestingly, patients with severe manifestations had a slightly shorter average hospitalisation duration of 16.0 days.

### 3.2. Self-Evaluation Scale Analysis

**Figure 1:**
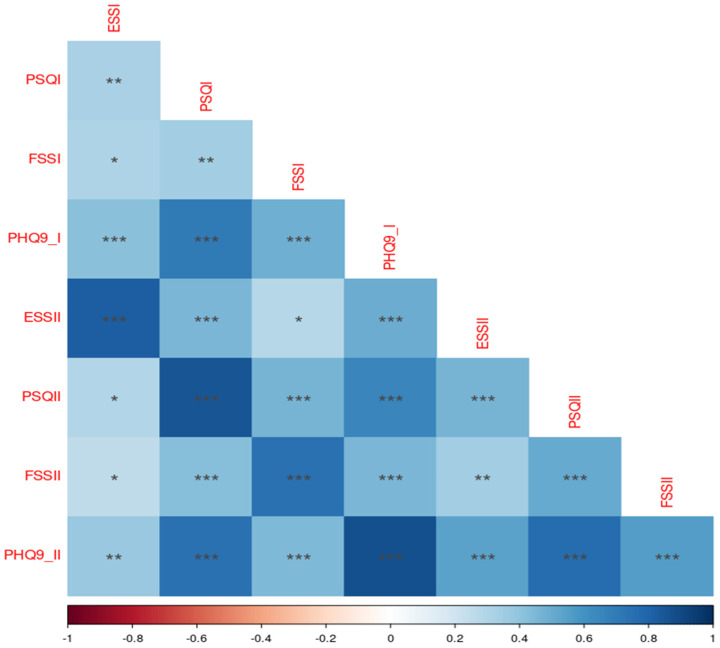
Correlation between self-evaluation scales. * *p* < 0.05; ** *p* < 0.01; *** *p* < 0.001.

Spearman correlation analysis revealed a strong and statistically significant correlation between all self-evaluation scales used in this study (*p* < 0.001 to *p* = 0.041), suggesting a consistent relationship between measures of sleep quality, daytime sleepiness, depression, and fatigue among all participants (Figure 1). Cronbach’s alpha values for the scales in our sample were as follows: ESS—0.85; PSQI—0.79; PHQ-9—0.86 and FSS—0.92. These values indicate good to excellent internal consistency for each scale.

These scales were compared between home-treated and hospital-treated participants (Table 3). Three months after confirmed infection, PHQ-9 scores showed a statistically significant difference (*p* < 0.001) between the home-treated and hospital-treated groups. Only 30.6% (*n* = 11) of participants treated at home reported minimal or no depressive symptoms, compared to 83.3% (*n* = 25) of participants treated in the hospital. Similarly, at six months after infection, significant disparities persisted in PHQ-9 scores (*p* < 0.001), with 30.6% (*n* = 11) and 90.0% (*n* = 27) reporting minimal or no symptoms in the home-treated and hospital-treated patient groups, respectively. For PSQI scores at three months, a substantial discrepancy was evident (*p* < 0.001), with 36.1% (*n* = 13) of the participants treated at home experiencing good sleep quality compared to 80.0% (*n* = 24) of the participants treated at the hospital. At six months, the difference remained significant (*p* = 0.015), with 47.2% (*n* = 17) and 76.7% (*n* = 23) reporting good sleep quality in the home and hospital groups, respectively.

When comparing results after three and six months, the total score on each self-evaluation scale was the same, and the *p*-value ranged from 0.103 to 0.640.

## 4. Discussion

The results of this study provide valuable insights into the impact of COVID-19 on various aspects of health and general well-being, including sleep quality, daytime sleepiness, depression, and fatigue among hospital-treated and home-treated individuals. Although quality of life was not directly assessed in this study, the validated instruments used, such as the PSQI, PHQ-9, and FSS, offer valuable insights into crucial aspects of well-being that significantly contribute to an individual’s overall quality of life. By examining sleep quality, depressive symptoms, and fatigue, we can indirectly discuss the impact of SARS-CoV-2 infection on essential dimensions of physical, mental, and emotional health and provide a more comprehensive understanding of the potential long-term consequences of COVID-19 on patients’ lives. These findings align with previously published articles indicating that COVID-19 is associated with poor sleep quality, early morning awakening, and daytime sleepiness [22].

Furthermore, the study corroborates the notion that patients who had previously been diagnosed positive for COVID-19 had a higher rate of depression than individuals in the general population [23], and that fatigue is also a common symptom after COVID-19 [24,25]. Many studies comparing hospitalised and non-hospitalised patients did not detect a significant difference in fatigue when comparing these groups [26,27,28]. In this study, 33.3% (*n* = 12) home-treated individuals compared to 53.3% (*n* = 16) hospital-treated patients suffered from fatigue 3 months after being diagnosed with SARS-CoV-2 infection and 56.7% (*n* = 17) patients six months after were suffering from fatigue. These results were not statistically significant, but it can be seen that participants who were hospitalised had higher fatigue prevalence.

In 2021, Ceban et al. published a meta-analysis comprising 74 studies that analysed fatigue and cognitive impairment in patients with post-COVID-19 syndrome [24]. They concluded that persistent fatigue and cognitive impairment after SARS-CoV-2 infection are common findings, as 22% of their study population demonstrated cognitive decline 12 or more weeks after being tested positive for COVID-19 disease [24].

According to the data available in the literature, poor sleep quality and depression symptoms are common findings in PCC patients [3,29]. The findings of the study conducted by Carnes et al., 2023, noted a significant correlation between the severity of PCC and the PSQI index. However, patients with PCC admitted to intensive care units and those treated at home demonstrated lower subjective sleep quality and shorter sleep time compared to healthy control groups, but not compared to hospitalised PCC groups [3].

This study revealed a statistically significant difference in PHQ-9 and PSQI scores between individuals treated at home and those who were hospitalised, meaning that individuals who were infected by SARS-CoV-2 and who had milder symptoms and were therefore treated at home had more severe depressive symptoms and worse sleep quality comparing to those with the more severe path of COVID-19. This suggests an important influence of the severity of COVID-19 and the treatment setting for the patient’s mental health and quality of sleep. These findings are similar to the findings of some studies published so far. For instance, Mazza et al., 2020, observed that outpatients showed increased anxiety and sleep disturbances, while the duration of hospitalisation was inversely correlated with posttraumatic stress disorder, depression, anxiety, and obsessive–compulsive symptoms. Furthermore, in the study published by Mazza et al., 2020 interviews were conducted by well-trained psychiatrists [29], proving self-questionnaires as valuable tools for assessing patient well-being. However, in Mazza et al.’s 2020 study participants were observed one month after infection and did not look at sleep quality rates. This study is unique in its long-term examination of depression, fatigue, and sleep quality indicators—key determinants of overall well-being. Notably, our findings demonstrate that individuals treated in a hospital setting with a more severe path of SARS-CoV-2 infection exhibit better outcomes across all these measures compared to those treated at home. Additionally, we found an interesting correlation that hospitalised patients with severe manifestations had a shorter average hospitalisation duration of 16.0 days compared to 18.33 days for patients with a moderately severe disease course.

The limitations of this study included a small cohort of patients and disparities in age and gender because of many restrictions that were in a pandemic, limited availability of patients and rapid decrease in people infected by the SARS-CoV-2 virus a few months after the start of the study. This study did not include the non-exposed control group, providing no basis for comparison of whether the pandemic increased scores or was due solely to COVID-19 infection.

More research with larger and more diverse samples is warranted to validate these findings and further explore the long-term effects of COVID-19 on sleep quality, daytime sleepiness, depression, and fatigue. Future research could replicate this study across the Baltic states to expand the scope and further validate the findings. A larger, multi-national sample would provide greater statistical power for identifying subtle effects and allow for regional cross-cultural comparisons. Additionally, an expanded study population would enable a more comprehensive investigation into the influence of comorbidities, such as urinary dysfunction, on PCC. This is particularly relevant given recent research by Di Bello et al. (2023), which demonstrated that the co-occurrence of urinary dysfunction and excessive daytime sleepiness can exacerbate the risk of mental health issues [30].

Some publications have proven age to be an independent factor contributing to sleep impairment and mental health, as younger patients demonstrated higher levels of sleep disturbances and depression symptoms compared to older patients [31]. They found a strong statistically significant correlation between all self-evaluation scales used, suggesting a consistent relationship between measures of sleep quality, daytime sleepiness, depression, and fatigue among all participants. This finding underscores the interrelation of these health aspects and the need for a comprehensive approach to managing PCC.

The study findings also highlighted the importance of addressing sleep disturbances and mental health disorders among COVID-19 patients. Semyachkina-Glushkovskaya et al., 2021 suggest that sleep hygiene and quality of sleep should be incorporated into rehabilitating patients with COVID-19 [13]. Targeted interventions and support strategies for patients treated at home can be essential to prevent adverse outcomes and improve the patient’s overall well-being.

Hawke et al., 2022, published a systematic review of various interventions for mental health, cognition, and psychological well-being in long-term COVID-19, enrolling 42 different trials, mostly randomised. They highlighted that the current scope of the associated intervention research is limited and still in progress [32].

Another study published in 2021 addressed non-drug interventions, proving they can reduce anxiety and depression scores and improve sleep quality among COVID-19 patients [33]. This study indicates that muscle relaxation, respiratory muscle-related rehabilitation training, Chinese medicine nursing technology, and internet-based self-help intervention could help improve mental health and general well-being.

## 5. Conclusions

In conclusion, this longitudinal cohort study marks the impact of SARS-CoV-2 infection on various aspects of health and general well-being, including sleep quality, daytime sleepiness, depression, and fatigue among hospital-treated and home-treated individuals. The study revealed significant disparities in sleep quality and depressive symptoms between home-treated and hospitalised patients, highlighting the influence of disease severity and treatment setting on long-term outcomes. Home-treated patients reported higher levels of depression and poorer sleep quality, while hospitalised patients demonstrated lower levels of depressive symptoms. These findings emphasise the importance of addressing sleep disturbances and mental health disorders in COVID-19 patients, particularly those treated at home. Recognizing the interrelations of sleep quality, daytime sleepiness, depression, and fatigue, and a comprehensive approach to managing PCC is crucial. Incorporating targeted interventions and support strategies for patients treated at home may be essential in addressing adverse outcomes and improving overall patient well-being.

## Figures and Tables

**Table 1:** Patient demographics.

Variable	Total	Home-Treated	Hospital-Treated
Demographics			
Age (years) *	44.05 ± 21.61	29.42 ± 7.68	61.60 ± 19.79
BMI (kg/m^2^)	25.79 ± 5.84	23.87 ± 5.17	28.10 ± 5.85
Male *	25.8% (*n* = 17)	5.6% (*n* = 2)	50.0% (*n* = 15)
Female *	74.2% (*n* = 49)	94.4% (*n* = 34)	50.0% (*n* = 15)
Smoking	28.8% (*n* = 19)	27.8% (*n* = 10)	30.0% (*n* = 9)
Second SARS-CoV-2 infection *	16.67% (*n* = 11)	19.4% (*n* = 7)	13.3% (*n* = 4)
Comorbidities			
Arterial hypertension *	24.2% (*n* = 16)	-	53.3% (*n* = 16)
Chronic heart failure *	16.7% (*n* = 11)	2.8% (*n* = 1)	33.3% (*n* = 10)
Diabetes mellitus *	13.6% (*n* = 9)	-	30.0% (*n* = 9)
Coronary heart disease *	10.6% (*n* = 7)	-	23.3% (*n* = 7)
Bronchial asthma *	3% (*n* = 2)	2.8% (*n* = 1)	3.3% (*n* = 1)
Chronic renal failure *	6.1% (*n* = 4)	-	13.3% (*n* = 4)
Cancer *	6.1% (*n* = 4)	-	13.3% (*n* = 4)

BMI: body mass index. * participants sociodemographic parameters and previous illnesses.

**Table 2:** Treatment and prevention of the SARS-CoV-2 infection.

Variable	Home-Treated, 54.5% (*n* = 36)	Hospital-Treated, 45.5% (*n* = 30)		
		Moderate, 21.2% (*n* = 14)	Moderately Severe, 18.2% (*n* = 12)	Severe, 6.1% (*n* = 4)
Oxygen therapy				
Not needed	100% (*n* = 36)	92.9% (*n* = 13)	25.0% (*n* = 3)	25.0% (*n* = 1)
FMR	-	7.1% (*n* = 1)	58.3% (*n* = 7)	-
HFNC	-	-	16.7% (*n* = 2)	25.0% (*n* = 1)
NIV	-	-	-	50.0% (*n* = 2)
Remdesivir therapy	-	-	8.3% (*n* = 1)	50.0% (*n* = 2)
Unvaccinated	2.8% (*n* = 1)	28.6% (*n* = 4)	58.3% (*n* = 7)	75% (*n* = 3)

FMR: face mask with reservoir; HFNC: high flow nasal cannula; NIV: non-invasive ventilation.

**Table 3:** Difference in the result of the self-evaluation scale between participants treated at home and hospital.

Variable	Home-Treated (*n* = 36)	Hospital-Treated (*n* = 30)	*p*-Value
PHQ-9 3 months after SARS-CoV-2 infection			**<0.001**
Minimal or none (0–4)	30.6% (*n* = 11)	83.3% (*n* = 25)	
Mild (5–9)	30.6% (*n* = 11)	13.3% (*n* = 4)	
Moderate (10–14)	13.9% (*n* = 5)	3.3% (*n* = 1)	
Moderately severe (15–19)	11.1% (*n* = 4)	-	
Severe (20–27)	13.9% (*n* = 5)	-	
PHQ-9 6 months after SARS-CoV-2 infection			**<0.001**
Minimal or none (0–4)	30.6% (*n* = 11)	90.0% (*n* = 27)	
Mild (5–9)	30.6% (*n* = 11)	6.7% (*n* = 2)	
Moderate (10–14)	22.2% (*n* = 8)	3.3% (*n* = 1)	
Moderately severe (15–19)	13.9% (*n* = 5)	-	
Severe (20–27)	2.8% (*n* = 1)	-	
ESS 3 months after SARS-CoV-2 infection			0.215
Lower normal daytime sleepiness (0–5)	50.0% (*n* = 18)	76.7% (*n* = 23)	
Higher normal daytime sleepiness (6–10)	25.0% (*n* = 9)	16.7% (*n* = 5)	
Mild excessive daytime sleepiness (11–12)	8.3% (*n* = 3)	3.3% (*n* = 1)	
Moderate excess daytime sleepiness (13–15)	8.3% (*n* = 3)	3.3% (*n* = 1)	
Severe excessive daytime sleepiness (16–24)	8.3% (*n* = 3)	-	
ESS 6 months after SARS-CoV-2 infection			0.163
Lower normal daytime sleepiness (0–5)	50.0% (*n* = 18)	73.3% (*n* = 22)	
Higher normal daytime sleepiness (6–10)	25.0% (*n* = 9)	10.0% (*n* = 6)	
Mild excessive daytime sleepiness (11–12)	8.3% (*n* = 3)	6.7% (*n* = 2)	
Moderate excess daytime sleepiness (13–15)	5.6% (*n* = 2)	-	
Severe excessive daytime sleepiness (16–24)	11.1% (*n* = 4)	-	
PSQI 3 months after SARS-CoV-2 infection			**<0.001**
Good sleep quality (0–5)	36.1% (*n* = 13)	80.0% (*n* = 24)	
Poor sleep quality (6–21)	63.9% (*n* = 23)	20.0% (*n* = 6)	
PSQI 6 months after SARS-CoV-2 infection			**0.015**
Good sleep quality (0–5)	47.2% (*n* = 17)	76.7% (*n* = 23)	
Poor sleep quality (6–21)	52.8% (*n* = 19)	23.3% (*n* = 7)	
FSS 3 months after SARS-CoV-2 infection			0.102
Not suffering from fatigue (0–35)	66.7% (*n* = 24)	46.7% (*n* = 14)	
Suffering from fatigue (36–63)	33.3% (*n* = 12)	53.3% (*n* = 16)	
FSS 6 months after SARS-CoV-2 infection			0.057
Not suffering from fatigue (0–35)	66.7% (*n* = 24)	43.3% (*n* = 13)	
Suffering from fatigue (36–63)	33.3% (*n* = 12)	56.7% (*n* = 17)	

Statistically significant (*p* < 0.05) difference highlighted in bold.

## Data Availability

The data presented in this study are available from the corresponding author upon request.

## References

[1] Munteanu I., Marc M., Gheorghevici C., Diaconu G.A., Feraru N., Sion D., Nemes R.M., Mahler B. (2023). Sleep quality aspects in Post-COVID-19 patients. J. Pers. Med..

[2] Soriano J.B., Murthy S., Marshall J.C., Relan P., Diaz J.V. (2022). A clinical case definition of post-COVID-19 condition by a Delphi consensus. Lancet. Infect. Dis./Lancet. Infect. Dis..

[3] Carnes-Vendrell A., Piñol-Ripoll G., Ariza M., Cano N., Segura B., Junque C., Béjar J., Barrue C., Garolera M., Arauzo V. (2024). Sleep quality in individuals with post-COVID-19 condition: Relation with emotional, cognitive and functional variables. Brain Behav. Immunity. Health.

[4] Bourmistrova N.W., Solomon T., Braude P., Strawbridge R., Carter B. (2022). Long-term effects of COVID-19 on mental health: A systematic review. J. Affect. Disord..

[5] Vindegaard N., Benros M.E. (2020). COVID-19 pandemic and mental health consequences: Systematic review of the current evidence. Brain Behav. Immun..

[6] De Sousa Martins E Silva E., Ono B.H.V.S., Souza J.C. (2020). Sleep and immunity in times of COVID-19. Rev. Da Assoc. Médica Bras..

[7] Pakpour A.H., Griffiths M.D., Ohayon M.M., Broström A., Lin C.-Y. (2020). Editorial: A Good Sleep: The Role of Factors in Psychosocial Health. Front. Neurosci..

[8] Sun X., Liu B., Liu S., Wu D.J.H., Wang J., Qian Y., Ye D., Mao Y. (2022). Sleep disturbance and psychiatric disorders: A bidirectional Mendelian randomisation study. Epidemiol. Psychiatr. Sci..

[9] Kaur T., Ranjan P., Chakrawarty A., Kasi K., Berry P., Suryansh S., Mazumder A., Khan M., Upadhyay A.D., Kaloiya G. (2021). Association of sociodemographic parameters with depression, anxiety, stress, sleep quality, Psychological Trauma, Mental Well-Being, and Resilience during the second wave of COVID-19 Pandemic: A Cross-Sectional Survey from India. Curēus.

[10] Sher L. (2020). COVID-19, anxiety, sleep disturbances and suicide. Sleep Med..

[11] Samushiya M.A., Kryzhanovsky S.M., Ragimova A.A., Berishvili T.Z., Chorbinskaya S.A., Ivannikova E.I. (2022). Psychoemotional Disorders and Sleep Impairments in Patients with COVID-19. Neurosci. Behav. Physiol..

[12] Al-Ameri L.T., Hameed E.K., Maroof B.S. (2022). Sleep quality in COVID-19 recovered patients. Sleep Sci..

[13] Semyachkina-Glushkovskaya O., Mamedova A., Vinnik V., Klimova M., Saranceva E., Ageev V., Yu T., Zhu D., Penzel T., Kurths J. (2021). Brain mechanisms of COVID-19-Sleep disorders. Int. J. Mol. Sci..

[14] Cellini N., Canale N., Mioni G., Costa S. (2020). Changes in sleep pattern, sense of time and digital media use during COVID-19 lockdown in Italy. J. Sleep Res..

[15] Mazza C., Ricci E., Biondi S., Colasanti M., Ferracuti S., Napoli C., Roma P. (2020). A Nationwide Survey of Psychological Distress among Italian People during the COVID-19 Pandemic: Immediate Psychological Responses and Associated Factors. Int. J. Environ. Res. Public Health.

[16] Rogers J.P., Chesney E., Oliver D., Pollak T.A., McGuire P., Fusar-Poli P., Zandi M.S., Lewis G., David A.S. (2020). Psychiatric and neuropsychiatric presentations associated with severe coronavirus infections: A systematic review and meta-analysis with comparison to the COVID-19 pandemic. Lancet Psychiatry.

[17] Richter K., Kellner S. (2021). “Coronasomnia”—Resilienzförderung durch Insomniebehandlung. Somnologie.

[18] Johns M.W. (1991). A new method for measuring daytime sleepiness: The Epworth Sleepiness Scale. Sleep.

[19] Buysse D.J., Reynolds C.F., Monk T.H., Berman S.R., Kupfer D.J. (1989). The Pittsburgh sleep quality index: A new instrument for psychiatric practice and research. Psychiatry Res..

[20] Vrublevska J., Trapencieris M., Rancans E. (2017). Adaptation and validation of the Patient Health Questionnaire-9 to evaluate major depression in a primary care sample in Latvia. Nord. J. Psychiatry.

[21] Valko P.O., Bassetti C.L., Bloch K.E., Held U., Baumann C.R. (2008). Validation of the fatigue severity scale in a Swiss cohort. Sleep.

[22] Alimoradi Z., Broström A., Tsang H.W.H., Griffiths M.D., Haghayegh S., Ohayon M.M., Lin C.-Y., Pakpour A.H. (2021). Sleep problems during COVID-19 pandemic and its’ association to psychological distress: A systematic review and meta-analysis. EClinicalMedicine.

[23] (2022). Mental Health and COVID-19: Early Evidence of the Pandemic’s Impact: Scientific Brief. https://iris.who.int/handle/10665/352189.

[24] Ceban F., Ling S., Lui L.M.W., Lee Y., Gill H., Teopiz K.M., Rodrigues N.B., Subramaniapillai M., Di Vincenzo J.D., Cao B. (2022). Fatigue and cognitive impairment in Post-COVID-19 Syndrome: A systematic review and meta-analysis. Brain Behav. Immun..

[25] Bansal A.S., Bradley A.S., Bishop K.N., Kiani-Alikhan S., Ford B. (2012). Chronic fatigue syndrome, the immune system and viral infection. Brain Behav. Immun..

[26] Jacobson K.B., Rao M., Bonilla H., Subramanian A., Hack I., Madrigal M., Singh U., Jagannathan P., Grant P. (2021). Patients With Uncomplicated Coronavirus Disease 2019 (COVID-19) Have Long-Term Persistent Symptoms and Functional Impairment Similar to Patients with Severe COVID-19: A Cautionary Tale During a Global Pandemic. Clin. Infect. Dis. (Online. Univ. Chicago Press).

[27] Abdallah S.J., Voduc N., Corrales-Medina V.F., McGuinty M., Pratt A., Chopra A., Law A., Garuba H.A., Thavorn K., Reid R.E.R. (2021). Symptoms, Pulmonary Function, and Functional Capacity Four Months after COVID-19. Ann. Am. Thorac. Soc..

[28] Menges D., Ballouz T., Anagnostopoulos A., Aschmann H.E., Domenghino A., Fehr J.S., Puhan M.A. (2021). Burden of post-COVID-19 syndrome and implications for healthcare service planning: A population-based cohort study. PLoS ONE.

[29] Mazza M.G., De Lorenzo R., Conte C., Poletti S., Vai B., Bollettini I., Melloni E.M.T., Furlan R., Ciceri F., Rovere-Querini P. (2020). Anxiety and depression in COVID-19 survivors: Role of inflammatory and clinical predictors. Brain Behav. Immun..

[30] Di Bello F., Scandurra C., Muzii B., Ruvolo C.C., Califano G., Mocini E., Creta M., Napolitano L., Morra S., Fraia A. (2023). Are excessive daytime sleepiness and lower urinary tract symptoms the triggering link for mental imbalance? An exploratory post hoc analysis. J. Clin. Med..

[31] Wang C., Pan R., Wan X., Tan Y., Xu L., McIntyre R.S., Choo F.N., Tran B., Ho R., Sharma V.K. (2020). A longitudinal study on the mental health of general population during the COVID-19 epidemic in China. Brain Behav. Immun..

[32] Hawke L.D., Nguyen A.T.P., Ski C.F., Thompson D.R., Ma C., Castle D. (2022). Interventions for mental health, cognition, and psychological wellbeing in long COVID: A systematic review of registered trials. Psychol. Med..

[33] Ding H., He F., Lu Y.-G., Hao S.-W., Fan X.-J. (2022). Effects of non-drug interventions on depression, anxiety and sleep in COVID-19 patients: A systematic review and meta-analysis. Eur. Rev. Med. Pharmacol. Sci..

